# Triangulating evidence for cardiometabolic Index: ROC cutoff, spline nonlinearity, and explainable machine learning for CVD high risk

**DOI:** 10.3389/fcvm.2026.1803704

**Published:** 2026-05-20

**Authors:** Haoran Wang, Zhiwei Huang, Jing Bai, Haiqin Yuan, Qiaotao Xie, Bing He, Li Guo, Li Wu, Dongliang Liu, Guirang Zhao, Jirui Cai, Jin Wang

**Affiliations:** 1 Luohe Central Hospital, Luohe Medical College, Luohe, China; 2 Henan University College of Medicine, Henan University, Kaifeng, China; 3 Huanghua People’s Hospital, Cangzhou, China; 4 Luohe Center for Disease Control and Prevention, Luohe, China

**Keywords:** cardiometabolic index, cardiovascular disease, interaction, machine learning, restricted cubic spline, risk stratification, SHAP, subgroup analysis

## Abstract

**Background:**

The cardiometabolic index (CMI), a composite marker reflecting central adiposity and dyslipidemia, may offer a pragmatic tool for community cardiovascular risk screening.

**Methods:**

We analyzed cross-sectional screening data from the ChinaHEART Luohe cohort. WHO chart-defined CVD high risk was a 10-year predicted risk ≥20%. CMI was evaluated per 1-unit increase and, for descriptive and stratified analyses, dichotomized using a rounded pragmatic analytic threshold of 0.7 derived from a crude ROC/Youden optimal threshold of 0.723. Discrimination (AUC with bootstrap 95% CIs), raincloud plots, restricted cubic splines (RCS), stepwise logistic regression, subgroup/interaction analyses, and an explainable ML pipeline (LASSO-random forest-SHAP) were applied.

**Results:**

Among 6,701 participants, 1,439 (21%) were classified as CVD high risk; prevalence was higher in the high-CMI group (≥0.7) than the low-CMI group (29% vs. 18%). CMI alone showed modest discrimination for WHO-defined CVD high-risk status (AUC: 0.571, 95% CI: 0.555–0.586), whereas multivariable models incorporating CMI showed higher discrimination (Model 3 AUC: 0.642, 95% CI: 0.626–0.659). The raincloud plot showed higher CMI in the high-risk group (*P* < 0.001), and RCS suggested nonlinearity (*P* for overall < 0.001; *P* for nonlinearity = 0.020). In Model 3, CMI was associated with higher odds of CVD high risk (OR: 1.31, 95% CI: 1.16–1.48 per 1-unit; OR: 1.50, 95% CI: 1.32–1.71 for ≥0.7 vs. <0.7). In ML, random forest achieved AUC 0.814; SHAP ranked CMI 3rd of 7 LASSO-selected features.

**Conclusions:**

Higher CMI was associated with WHO-defined CVD high-risk status with a nonlinear pattern and consistent importance across conventional and explainable ML analyses, supporting its potential utility as an adjunct screening marker.

## Introduction

Cardiovascular disease (CVD) remains a leading cause of morbidity and mortality worldwide, and prevention strategies increasingly emphasize early risk identification and targeted intervention (1, 2). In community screening settings, risk stratification tools that leverage routinely collected anthropometric and biochemical measures are particularly valuable because they are scalable and feasible in resource-variable environments.

Adiposity-related metabolic dysfunction and lipid abnormalities often co-occur and jointly accelerate cardiometabolic deterioration. Conventional single-domain markers [e.g., waist circumference or triglycerides (TG) alone] may not fully capture the combined burden of central adiposity and dyslipidemia. Waist-to-height ratio (WHtR) has been suggested as a simple and scalable indicator of central adiposity for cardiometabolic risk screening, with advantages in interpretability and comparability across body sizes (3–7). Accordingly, the cardiometabolic index (CMI), which integrates WHtR with an atherogenic lipid component (TG/HDL-C), has been proposed as a pragmatic composite marker that may reflect cardiometabolic derangements more comprehensively than isolated indicators (8–11).

While accumulating evidence suggests that composite metabolic indices are associated with hypertension, diabetes, and other cardiometabolic outcomes, less is known about whether CMI aligns with guideline-relevant cardiovascular risk categories in community-dwelling Chinese adults, particularly when CVD high-risk is defined by internationally recognized risk charts. Moreover, the dose–response pattern may be nonlinear, and the association may differ across subgroups characterized by age, lifestyle factors, and medication status—each of which has implications for clinical interpretation.

Therefore, using community screening data from the ChinaHEART Luohe cohort, we aimed to: (1) identify a ROC/Youden-derived optimal threshold for CMI and apply a rounded pragmatic analytic threshold for descriptive grouping and stratified analyses and evaluate its discrimination under stepwise adjustment; (2) examine the association between CMI and World Health Organization (WHO)-defined CVD high-risk status using logistic regression and restricted cubic splines; (3) explore subgroup heterogeneity and formal interaction; and (4) use machine-learning approaches [least absolute shrinkage and selection operator [LASSO], random forest [RF], Shapley additive explanations [SHAP]] to corroborate the importance of CMI in relation to CVD high-risk status.

## Methods

### Study design and population

This cross-sectional study used community screening data from the Luohe branch of the China Health Evaluation and risk Reduction through nationwide Teamwork (ChinaHEART) program in Henan Province, China, conducted between March 2021 and February 2022 (12, 13). Community residents were recruited through township hospitals. Eligible participants were adults aged 35–75 years who were permanent residents of the project area (≥6 months of residence in the preceding 12 months) and who provided written informed consent. The study was reported in accordance with the Strengthening the Reporting of Observational Studies in Epidemiology (STROBE) statement and conducted in line with the Declaration of Helsinki (14, 15). Ethical approval was obtained from the Ethics Committee of Fuwai Hospital (approval No. 2014–574) and filed with the local ethics committee of Luohe Central Hospital. A total of 6,860 participants were initially enrolled. For baseline descriptive analyses, we used a complete-case dataset requiring non-missing data for all variables presented in Table 1, yielding 6,701 participants. For regression-based analyses, including logistic regression, restricted cubic spline, subgroup, and interaction analyses, we used an analysis-specific complete-case approach requiring non-missing data for the exposure, outcome, and variables included in the corresponding model. Therefore, the effective sample size could vary across analyses. No *a priori* sample size calculation was performed because this study was a secondary cross-sectional analysis of an existing community-based screening cohort (the Luohe branch of the ChinaHEART program). All eligible participants with complete data during the study period were included in the present analysis.

**Table 1:** Baseline characteristics by CMI group using a rounded analytic threshold of 0.7.

Variable	Overall *N* = 6,701	Low CMI (<0.7) *N* = 4,375	High CMI (≥0.7) *N* = 2,326	*P*-value
Age (years)	58 (51, 66)	59 (50, 67)	58 (51, 66)	0.858
Sex (male)	2,518 (38%)	1,762 (40%)	756 (33%)	<0.001
Married	5,955 (89%)	3,909 (89%)	2,046 (88%)	0.094
Educated	5,439 (81%)	3,610 (83%)	1,829 (79%)	<0.001
Alcohol consumption	367 (5.5%)	217 (5.0%)	150 (6.4%)	0.013
Current smoking	1,336 (20%)	916 (21%)	420 (18%)	0.005
Systolic blood pressure (mmHg)	137 (126, 150)	135 (124, 148)	143 (131, 152)	<0.001
Diastolic blood pressure (mmHg)	83 (77, 91)	82 (76, 89)	86 (79, 95)	<0.001
Heart rate (bpm)	76 (70, 83)	75 (70, 82)	77 (71, 84)	<0.001
Fasting glucose (mmol/L)	5.40 (5.19, 6.00)	5.40 (5.10, 5.80)	5.70 (5.30, 6.40)	<0.001
Triglycerides (mmol/L)	1.52 (1.14, 2.03)	1.27 (1.02, 1.55)	2.33 (1.88, 3.02)	<0.001
HDL-C (mmol/L)	1.43 (1.23, 1.66)	1.52 (1.33, 1.75)	1.26 (1.11, 1.43)	<0.001
Non-HDL-C (mmol/L)	3.31 (2.66, 3.97)	3.12 (2.52, 3.71)	3.72 (3.03, 4.49)	<0.001
Hypertension	1,536 (23%)	788 (18%)	748 (32%)	<0.001
Diabetes mellitus	421 (6.3%)	211 (4.8%)	210 (9.0%)	<0.001
Stroke	183 (2.7%)	120 (2.7%)	63 (2.7%)	0.997
Family history of stroke	153 (2.3%)	82 (1.9%)	71 (3.1%)	0.003
CVD high-risk status	1,439 (21%)	770 (18%)	669 (29%)	<0.001
Antihypertensive drug therapy	883 (13%)	426 (9.7%)	457 (20%)	<0.001
Antidiabetic drug therapy	261 (3.9%)	133 (3.0%)	128 (5.5%)	<0.001
Statin use	188 (2.8%)	92 (2.1%)	96 (4.1%)	<0.001

Continuous variables: median (Q1, Q3). Dichotomous variables: *n* (%).

Mann–Whitney *U*-test; Pearson's Chi-squared test.

### Data collection and measurements

Data were collected by trained staff following standardized ChinaHEART procedures (12, 13). Face-to-face interviews using structured questionnaires were conducted to obtain sociodemographic characteristics (age, sex, marital status, and education), lifestyle factors (current smoking and alcohol consumption), medical history (hypertension, diabetes mellitus, and stroke), family history of stroke, and current medication use (antihypertensive drugs, antidiabetic drugs, and statins). Blood pressure (systolic and diastolic) and heart rate were measured using an electronic sphygmomanometer after at least 5 min of seated rest, with two readings recorded. After an overnight fast, venous blood samples were collected to measure fasting glucose and lipid parameters [triglycerides and high-density lipoprotein cholesterol (HDL-C)]; non-high-density lipoprotein cholesterol [non-HDL-C] was derived accordingly. Height and waist circumference were measured to compute waist-to-height ratio (WHtR) for calculating CMI.

### Exposure definitions: CMI

CMI was calculated as: CMI = (TG/HDL-C) × WHtR, where WHtR = waist circumference/height.

TG and HDL-C were fasting measurements (mmol/L), and waist circumference and height were measured in the same unit (m), so WHtR is dimensionless. CMI was analyzed (1) as a continuous variable per 1-unit increase and (2) as a categorical variable (high vs. low) using a rounded pragmatic analytic threshold derived from the Youden-index optimal threshold from the crude ROC analysis.

### Outcome definition: CVD high-risk status

CVD high-risk status was defined using the laboratory-based WHO CVD risk charts (16, 17). Participants with a 10-year predicted CVD risk ≥20% were classified as high risk; others were classified as non-high risk.

### Covariates

To maintain interpretability and avoid over-adjustment for factors directly embedded in the chart-based risk classification, we used stepwise models with prespecified covariates: Model 1 (crude): exposure only. Model 2: Model 1 + alcohol consumption and marital status (Married). Model 3: Model 2 + education, family history of stroke, antidiabetic drug therapy, antihypertensive drug therapy, and statin use.

### Statistical analysis

Continuous variables were summarized as median (Q1, Q3) and categorical variables as *n* (%). Group differences by CMI category were assessed using the Mann–Whitney *U*-test for continuous variables and Pearson's chi-squared test for categorical variables. Discrimination of CMI for WHO-defined CVD high-risk status was evaluated by ROC curves under crude and adjusted models, with area under the curve (AUCs) reported alongside bootstrap 95% confidence intervals; the Youden index from the crude ROC curve was used to derive the primary cutoff for CMI grouping. A raincloud plot was used to visualize the distribution of CMI by CVD high-risk status, complementing tabular summaries.

Associations between CMI and CVD high-risk status were quantified using logistic regression under stepwise adjustment (Models 1–3) for both continuous CMI (per 1-unit increase) and categorical CMI (high vs. low). Potential nonlinearity in the dose-response relationship was examined using restricted cubic splines in logistic models, with *P*-values reported for overall association and nonlinearity. Exploratory subgroup forest plots were generated using stratum-specific unadjusted logistic models for descriptive comparison across prespecified strata. Formal interaction was additionally evaluated using likelihood ratio tests comparing models with and without interaction terms. All analyses were performed in R (version 4.5.2), and two-sided *P* < 0.05 was considered statistically significant.

For the machine-learning (ML) framework, we used a parsimonious, clinically interpretable candidate feature set available in the baseline dataset, including age, sex, LDL-C, fasting glucose, current smoking, alcohol use, history of hypertension, history of diabetes, and CMI. We intentionally excluded triglycerides and non-HDL-C from the candidate pool to reduce redundancy and collinearity, because CMI is a composite cardiometabolic marker that already incorporates lipid-related and anthropometric information; including highly overlapping lipid metrics could lead to “double counting” and unstable feature attribution. Feature selection was performed using LASSO logistic regression with 10-fold cross-validation (glmnet), and the penalty parameter was chosen using the one-standard-error rule to favor a more parsimonious model. The variables retained by LASSO were then used to train a random forest classifier (randomForest) with a 70/30 train–test split, and model discrimination was evaluated by the AUC. To improve interpretability, we quantified feature contributions using SHAP values computed with fastshap, and summarized global importance by the mean absolute SHAP value (higher values indicate greater overall contribution to the predicted probability of CVD high-risk). As a supplementary *post hoc* sample size assessment, we estimated the required sample size for detecting the observed difference in the prevalence of WHO-defined CVD high-risk status between the high- and low-CMI groups using a two-sided *α* of 0.05, target powers of 80% and 90%, and the observed group allocation ratio. Because missingness differed across variables, baseline descriptive analyses and model-based analyses were conducted on analysis-specific complete-case datasets according to the variables required for each analysis.

## Results

### Participant characteristics

A total of 6,701 participants were included in the baseline analyses; 1,439 (21%) met the WHO-defined CVD high-risk criteria. Using the pragmatic cutoff applied for grouping (CMI ≥ 0.7), 2,326 (35%) were classified as high CMI. Compared with the low-CMI group, participants with high CMI had higher systolic/diastolic blood pressure, higher fasting glucose and triglycerides, lower HDL-C, and a higher prevalence of hypertension and diabetes, as well as a higher proportion of CVD high-risk status (29% vs. 18%; *P* < 0.001). Based on this observed between-group difference in WHO-defined CVD high-risk prevalence and the observed group allocation ratio, the estimated total sample size required for a two-sided *α* of 0.05 was 508 for 80% power and 681 for 90% power, both substantially lower than the actual analytic sample size of 6,701.

### ROC analysis and cutoff determination

**Figure 1:**
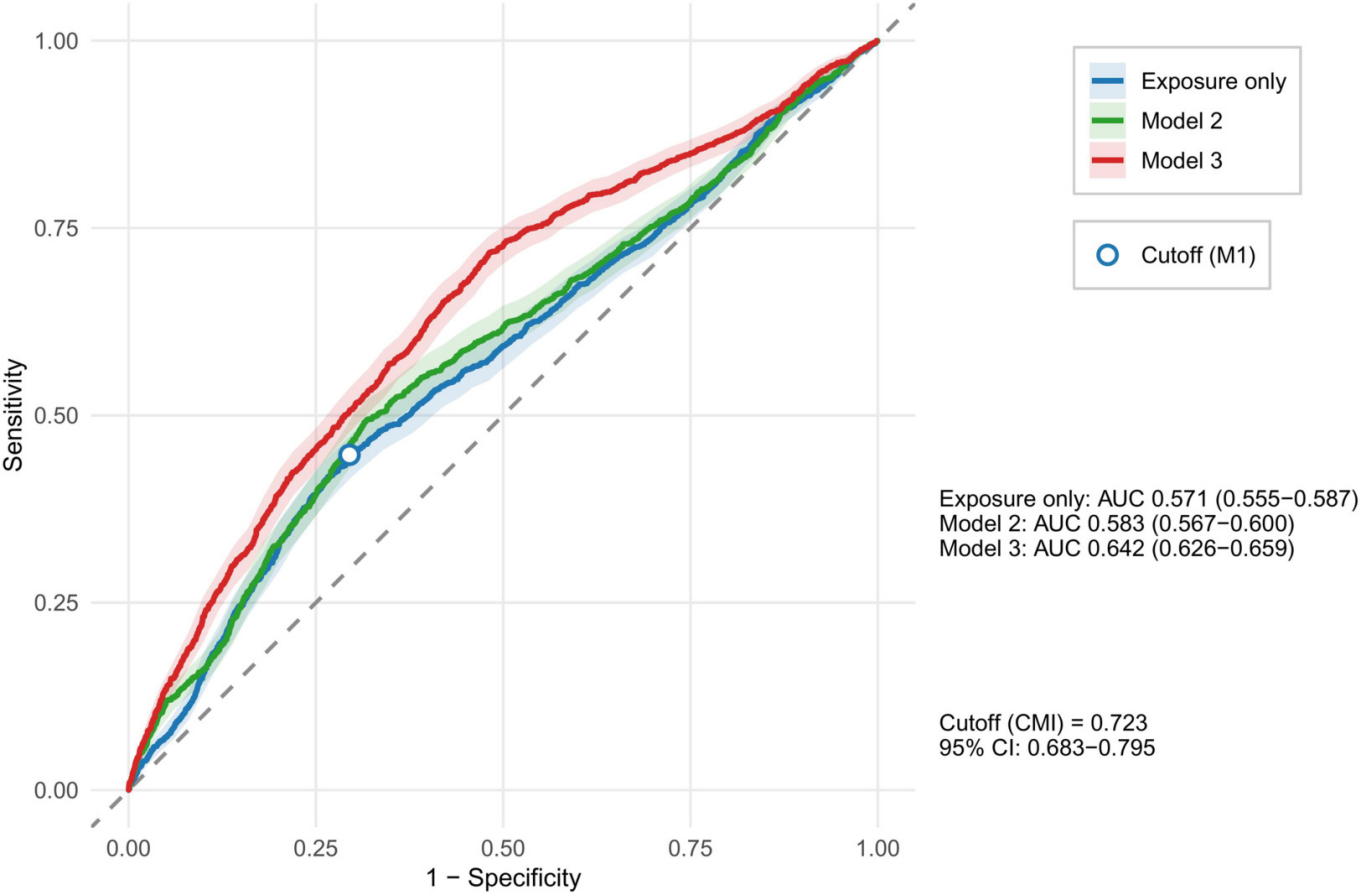
ROC curves and Youden-index cutoff for CMI. Receiver operating characteristic (ROC) curves for CMI alone in the crude model and for multivariable models incorporating CMI (Models 2 and 3). AUC (95% CI) for each model is reported. The Youden-index optimal cutoff for CMI derived from the crude model is indicated.

In ROC analysis, CMI alone showed modest discrimination for CVD high-risk status (AUC: 0.571). Higher AUCs were observed for multivariable models incorporating CMI, including Model 3 (AUC 0.642). The Youden-index optimal threshold for CMI was 0.723 (95% CI: 0.683–0.795). For practical presentation across baseline tables and stratified analyses, we used a rounded pragmatic analytic threshold of 0.7 to define high vs. low CMI (Figure 1).

### Distributional difference by outcome (raincloud plot)

**Figure 2:**
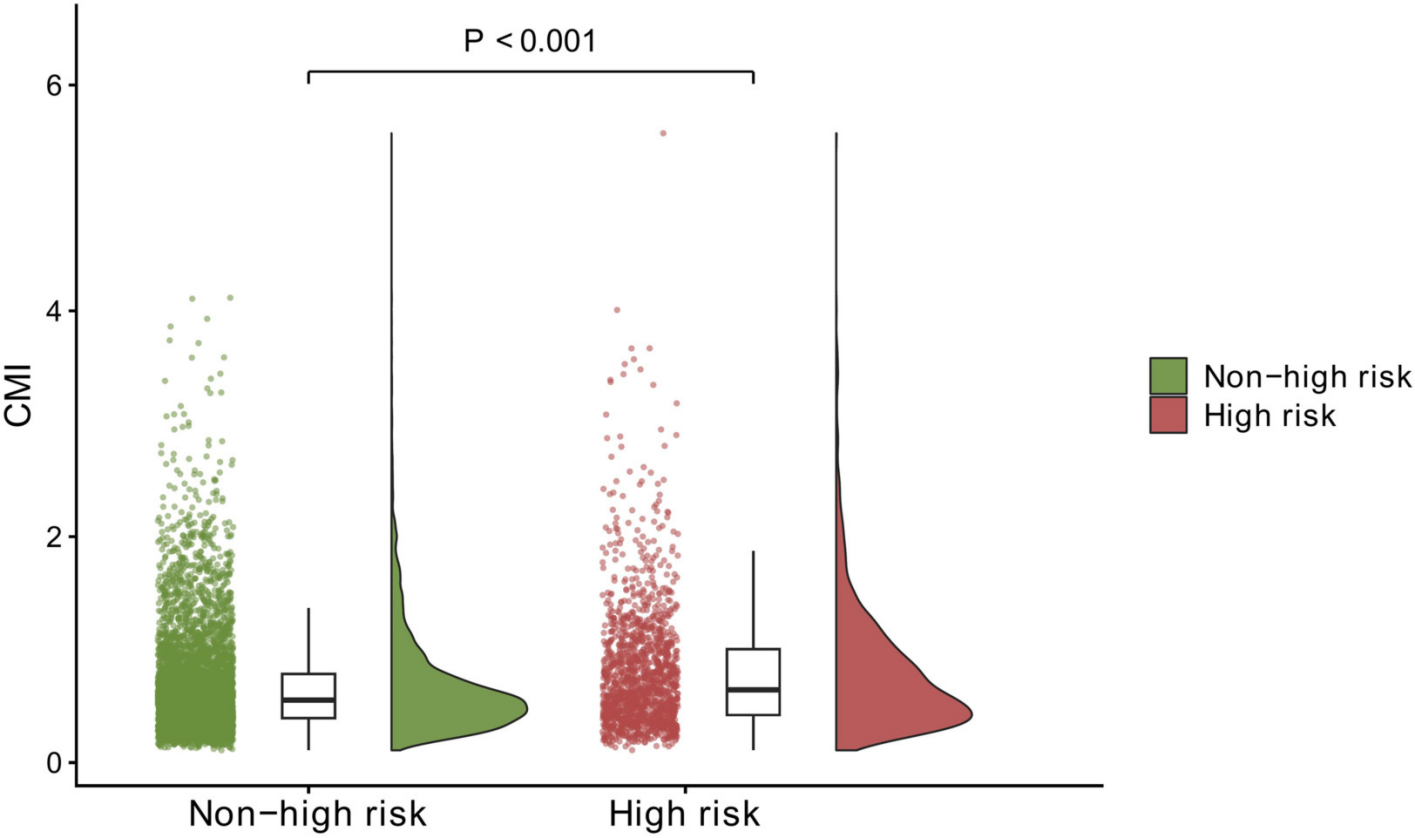
Raincloud plot of CMI by CVD high-risk status. Raincloud plot comparing CMI distributions between non-high-risk and high-risk groups. Points represent individual participants; boxplots summarize median and interquartile range; half-violin density displays distribution shape. *P*-value for between-group difference is shown.

The raincloud plot demonstrated a clear right-shift of CMI in participants classified as CVD high-risk, with a statistically significant between-group difference (*P* < 0.001) (Figure 2).

### Association between CMI and CVD high-risk status

In logistic regression, higher CMI was consistently associated with higher odds of CVD high-risk status (Table 2). For continuous CMI, the fully adjusted estimate (Model 3) was odds ratio (OR) 1.31 [95% confidence interval (CI) 1.16–1.48] per 1-unit increase (*P* < 0.001). When modeled categorically, high CMI (≥0.7) was associated with 50% higher odds of CVD high-risk compared with low CMI in Model 3 (OR: 1.50, 95% CI: 1.32–1.71; *P* < 0.001).

**Table 2:** Associations of CMI with CVD high-risk status.

Variable	Model 1		Model 2		Model 3	
	OR (95% CI)	*P*-value	OR (95% CI)	*P*-value	OR (95% CI)	*P*-value
CMI (per 1-unit increase)	1.61 (1.44, 1.80)	<0.001	1.60 (1.43, 1.79)	<0.001	1.31 (1.16, 1.48)	<0.001
CMI group (High vs. Low; cutoff=0.7)	1.86 (1.65, 2.09)	<0.001	1.84 (1.64, 2.07)	<0.001	1.50 (1.32, 1.71)	<0.001

Model 1: crude (exposure only). Model 2: adjusted for alcohol consumption and marital status. Model 3: additionally adjusted for education, family history of stroke, antidiabetic drug therapy, antihypertensive drug therapy, and statin use. Binary CMI group uses a rounded analytic threshold of 0.7 (high: CMI ≥0.7; low: CMI <0.7), derived from the Youden-index optimal threshold in the crude ROC analysis.

### Dose–response relationship (restricted cubic spline)

**Figure 3:**
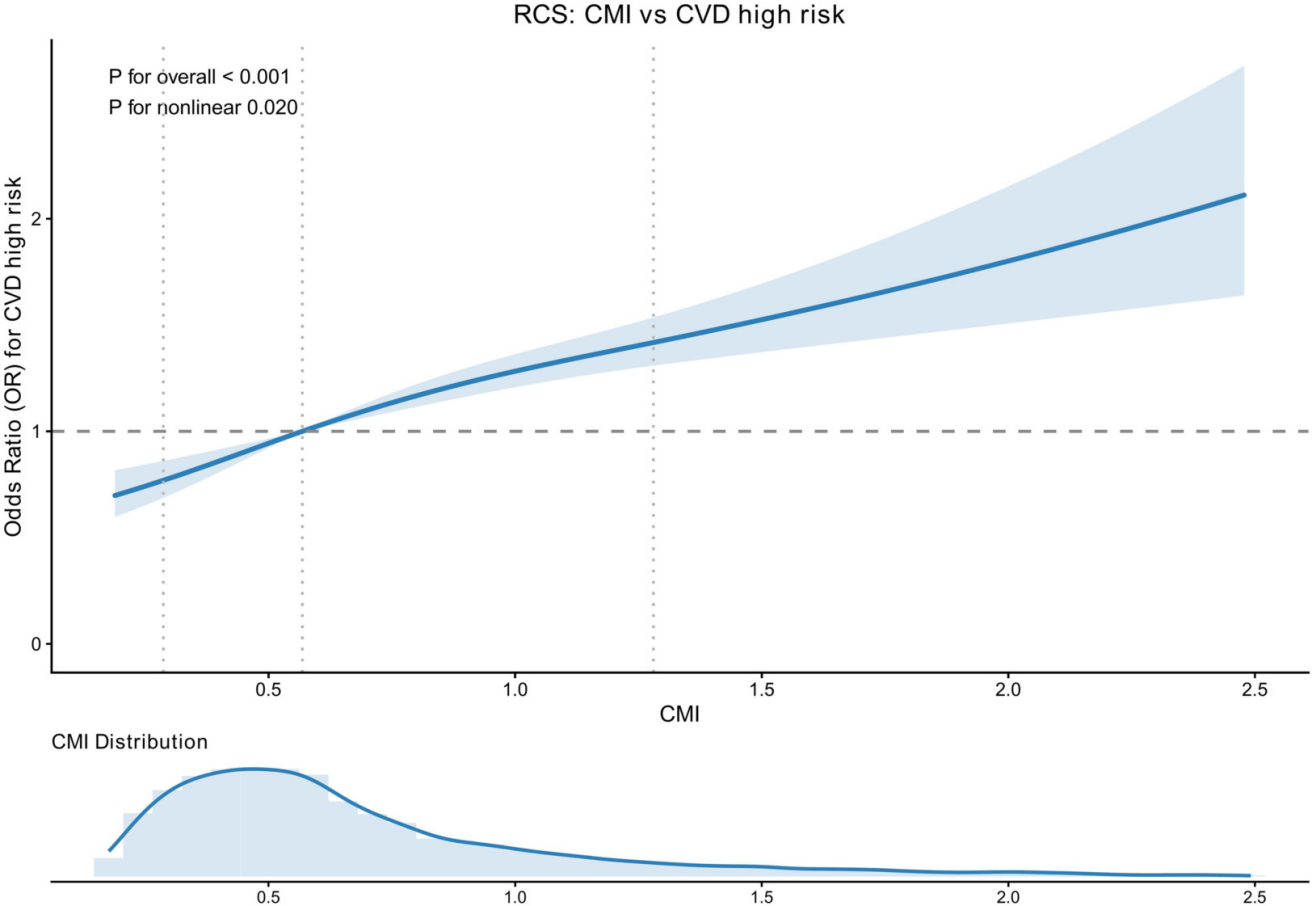
Restricted cubic spline analysis of CMI and CVD high-risk status. Restricted cubic spline showing the association between cardiometabolic index (CMI) and odds of WHO-defined CVD high-risk status. Solid line indicates adjusted odds ratio and shaded area indicates 95% confidence interval; *P* for overall and *P* for nonlinearity are shown.

Restricted cubic spline analysis suggested a significant nonlinear dose-response relationship between CMI and CVD high-risk status (*P* overall < 0.001; *P* for nonlinearity = 0.020) (Figure 3).

### Subgroup and interaction analyses

**Figure 4:**
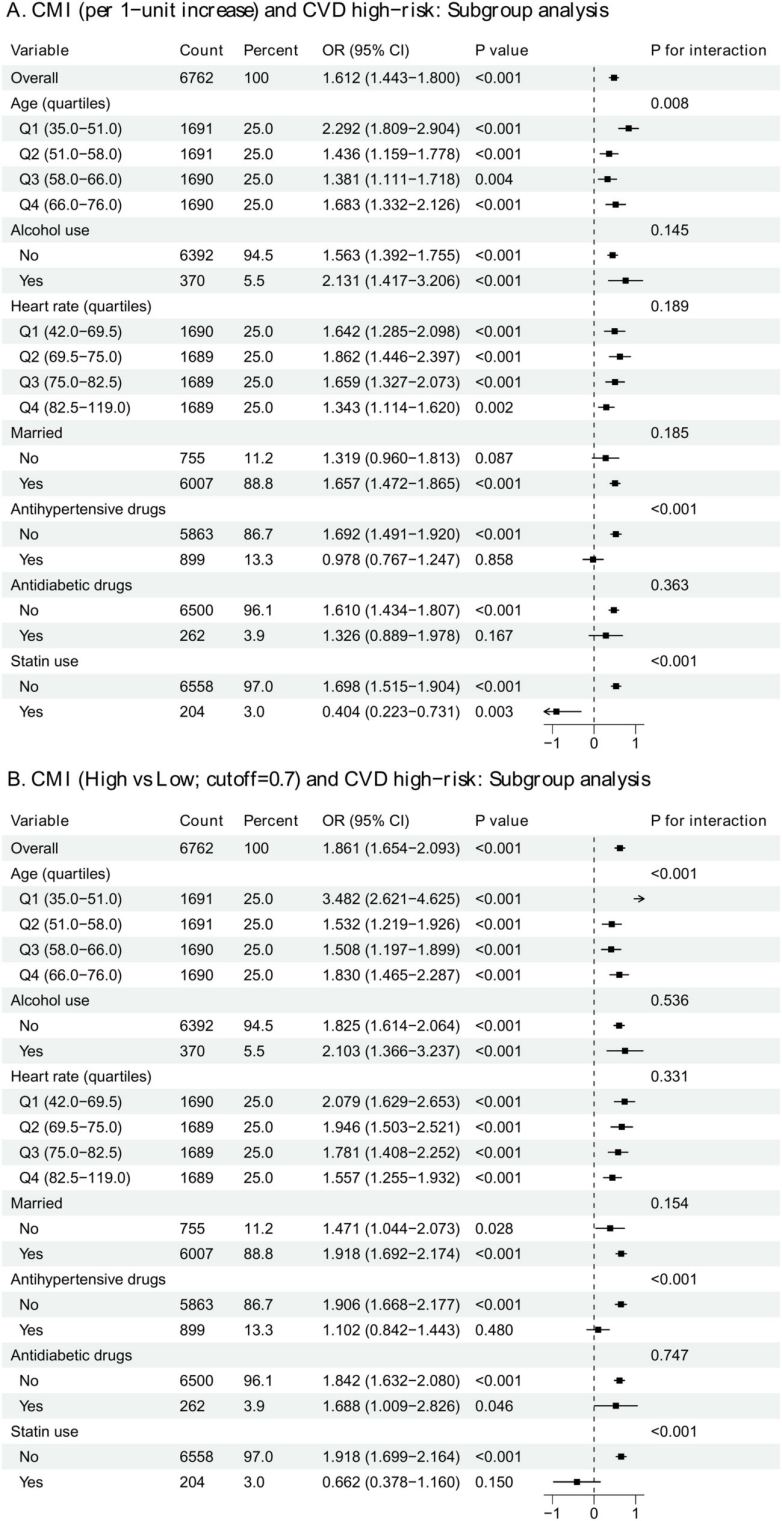
Subgroup analyses of CMI and CVD high-risk status. Exploratory unadjusted forest plots of the associations between CMI and CVD high-risk status across prespecified subgroups. Panel A: per 1-unit increase in CMI; Panel B: high vs. low CMI using the rounded analytic threshold of 0.7. Odds ratios (ORs) with 95% CIs are shown, along with *P* for interaction. Analyses were performed using the available complete cases for the exposure, outcome, and stratification variable within each subgroup; therefore, counts may differ from those reported in Table 1.

**Figure 5:**
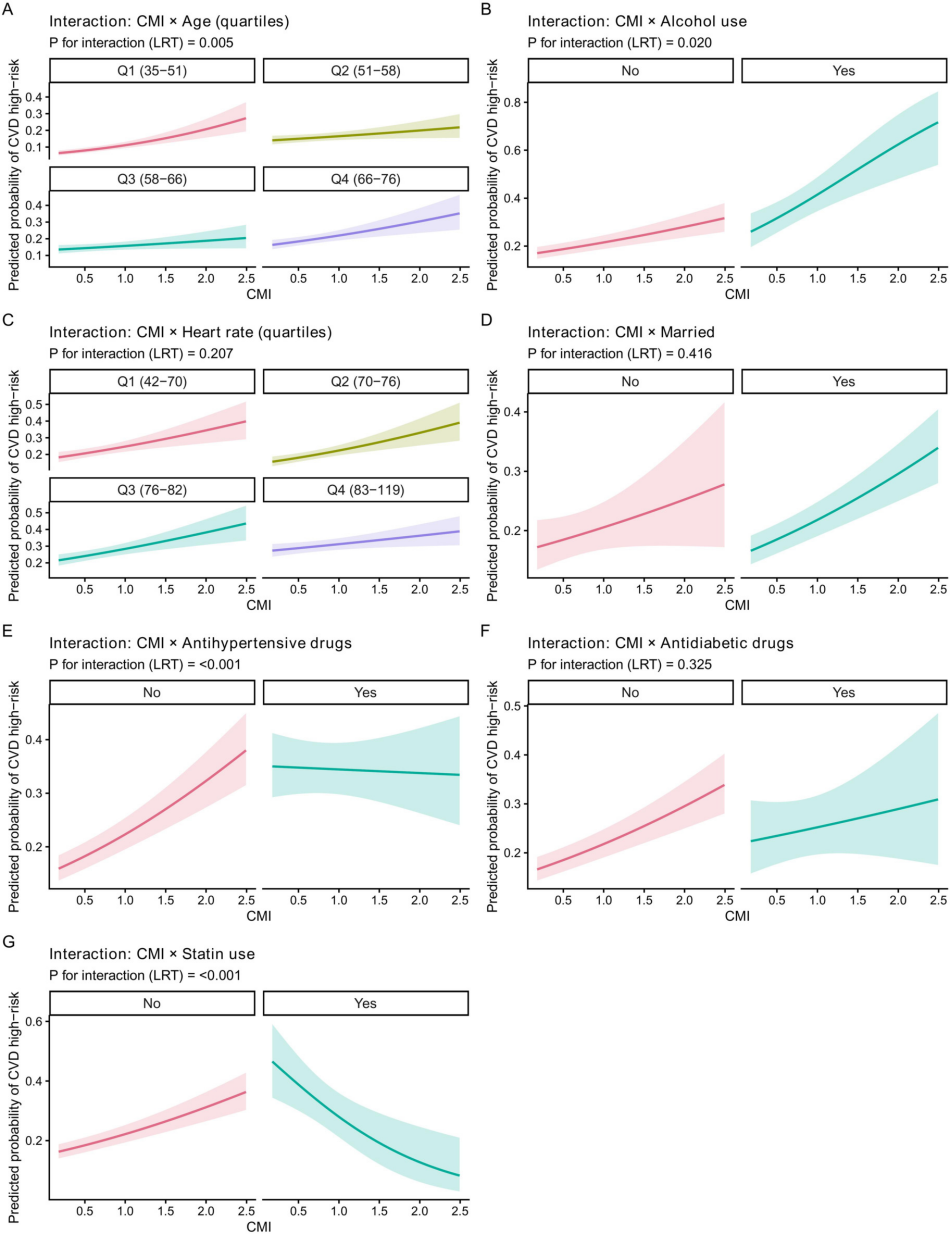
Interaction plots of CMI with subgroup factors. Predicted probability of CVD high-risk status across CMI levels stratified by **(A)** age quartiles, **(B)** alcohol use, **(C)** heart rate quartiles, **(D)** marital status, **(E)** antihypertensive drugs, **(F)** antidiabetic drugs, and **(G)** statin use. Shaded bands indicate 95% confidence intervals; *P* for interaction is from likelihood ratio tests.

In the exploratory unadjusted subgroup forest plots (Figure 4), the positive association between CMI and CVD high-risk status was broadly consistent across strata for continuous CMI (overall OR: 1.61, 95% CI: 1.44–1.80). Evidence of effect modification was observed for age (*P* for interaction = 0.008), antihypertensive drug use (*P* for interaction < 0.001), and statin use (*P* for interaction < 0.001), while interactions were not statistically significant for alcohol use, heart rate quartiles, marital status, or antidiabetic drugs in the subgroup panel. Interaction curves (Figure 5) were consistent with subgroup findings, showing significant heterogeneity for age (*P* = 0.005), alcohol use (*P* = 0.020), antihypertensive drugs (*P* < 0.001), and statin use (*P* < 0.001), whereas other interaction tests were not statistically significant. The subgroup forest plots were intended as exploratory descriptive analyses, whereas Figure 5 presents formal interaction curves and interaction testing.

### Exploratory machine learning interpretation (LASSO + RF + SHAP)

**Figure 6:**
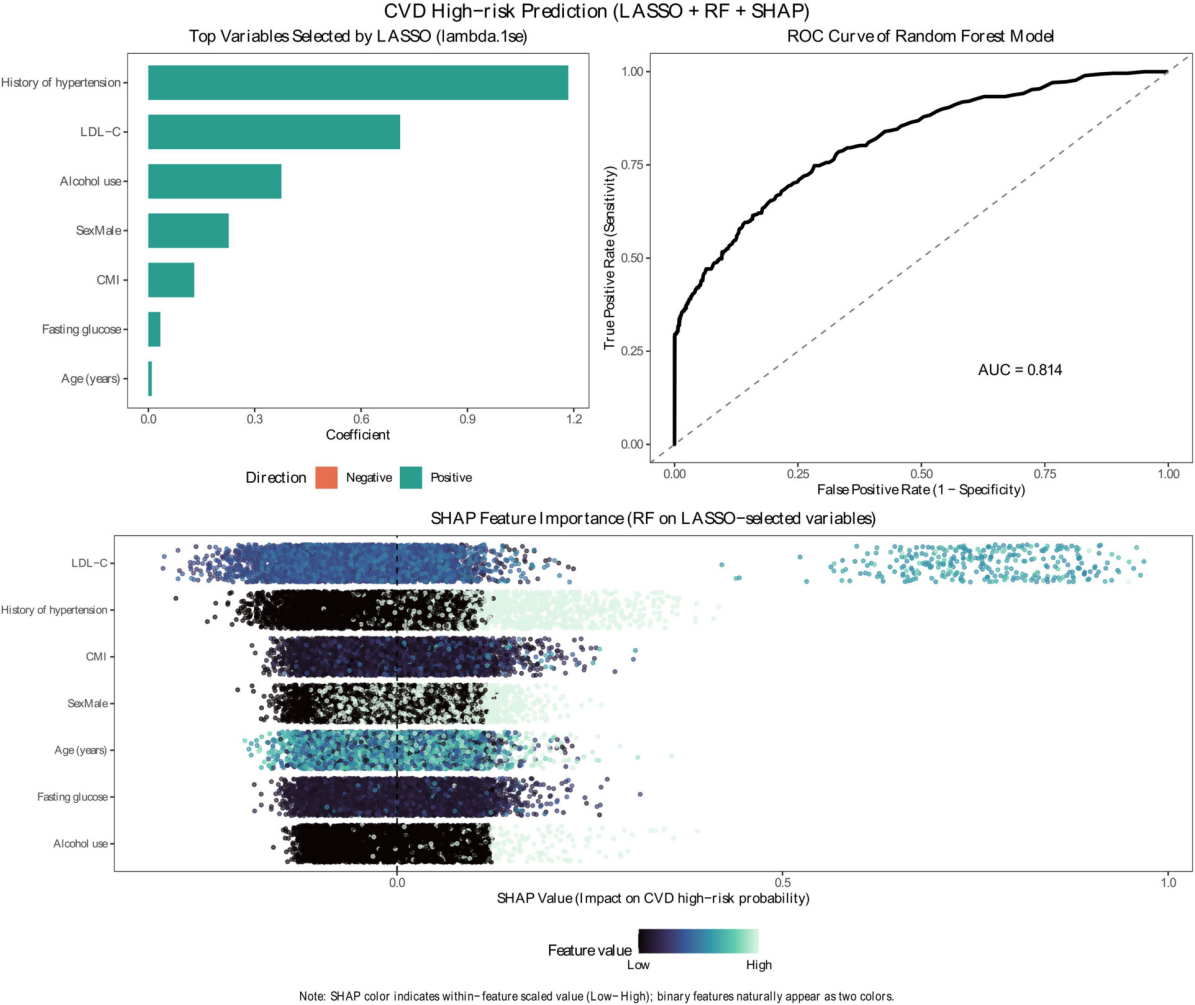
Explainable machine-learning validation (LASSO + RF + SHAP). Machine-learning pipeline for predicting CVD high-risk status. Left: LASSO-selected predictors (*λ*.1se) and coefficient direction. Middle: ROC curve of the random forest model (AUC shown). Bottom: SHAP beeswarm plot showing feature importance and direction of impact on predicted probability; color represents within-feature value (low to high).

Using the LASSO model with *λ*.1se, seven predictors were retained as the most informative variables for CVD high-risk classification (Figure 6). A Random Forest model trained on these LASSO-selected predictors achieved good discrimination on the test set (AUC = 0.814). SHAP analysis further quantified the relative importance of the selected predictors (rank x of y selected, where “y” denotes the number of LASSO-retained predictors). Specifically, SHAP ranked the predictors in the following descending order of importance: (1/7) LDL-C, (2/7) history of hypertension, (3/7) CMI, (4/7) male sex, (5/7) age, (6/7) fasting glucose, and (7/7) alcohol use. Across these predictors, higher LDL-C, the presence of hypertension, higher CMI, male sex, older age, higher fasting glucose, and alcohol use tended to shift SHAP values to the positive side, indicating higher predicted probability of being classified as CVD high-risk.

## Discussion

### Principal findings

In this large, community-based cross-sectional analysis from the ChinaHEART Luohe program, higher cardiometabolic index (CMI) was consistently associated with a higher likelihood of WHO chart–defined CVD high-risk status. In the analytic sample (*N* = 6,701), CVD high-risk prevalence was 21% overall and was higher among participants classified as high CMI (≥0.7) than low CMI (<0.7) (29% vs. 18%). The association remained evident under stepwise adjustment using prespecified covariates selected to preserve interpretability under chart-based risk classification.

### ROC-derived cutoff and pragmatic risk stratification

A key analytic step in this study was to first determine a data-driven cutoff for pragmatic grouping. Using the crude ROC curve, CMI alone showed modest discrimination for WHO-defined CVD high-risk status (AUC 0.571). Higher AUCs in Models 2 and 3 reflected the performance of multivariable models incorporating CMI rather than an intrinsic improvement in the discriminatory ability of CMI itself. The Youden-index optimal threshold from the crude model was 0.723 (95% CI: 0.683–0.795), and we used a rounded pragmatic analytic threshold (0.7) for consistent presentation across baseline comparison and stratified analyses; this threshold should not be interpreted as an established clinical cutoff. These findings suggest that CMI should be viewed as a practical screening marker rather than a standalone diagnostic tool: its single-index discrimination is limited, but it can support efficient stratification when combined with routinely available characteristics (1, 18, 19).

### Distributional separation (raincloud plot) supports the cutoff-based grouping

Beyond summary statistics, the raincloud plot provided an intuitive depiction of the between-group difference: CMI distribution was clearly right-shifted among participants classified as CVD high risk, with a statistically significant difference (*P* < 0.001). This visualization complements the ROC-derived cutoff logic by showing that the cutoff-based grouping is grounded in observable distributional separation rather than an arbitrary threshold choice.

### Dose–response pattern and nonlinearity (RCS)

The restricted cubic spline analysis further indicated that the CMI–risk relationship is not strictly linear. We observed a significant overall association with evidence of nonlinearity (*P* for overall < 0.001; *P* for nonlinearity = 0.020). From a clinical and public-health perspective, a nonlinear pattern implies that the incremental increase in risk associated with rising CMI may be disproportionate across different ranges, which is relevant when using a single cutoff for communication or triage: individuals near certain ranges of CMI may experience a steeper increase in predicted risk-category membership.

### Association estimates under stepwise adjustment

In logistic regression, the association was directionally consistent whether CMI was modeled continuously or categorically. In the fully adjusted model (Model 3), the odds of CVD high-risk status increased by ∼31% per 1-unit increase in CMI (OR: 1.307, 95% CI: 1.157–1.477), and high CMI (≥0.7) was associated with ∼50% higher odds compared with low CMI (OR: 1.504, 95% CI: 1.323–1.710). Importantly, because the outcome is a chart-defined risk category rather than adjudicated events, the interpretation is that CMI aligns with (and may help flag) individuals more likely to be classified as high risk by WHO charts—this is useful for screening workflows but requires prospective confirmation for incident outcomes (8–11, 20–23).

### Effect heterogeneity: subgroup and interaction interpretation

Subgroup analyses suggested that the positive CMI–risk association was broadly consistent across strata, but heterogeneity was observed for age quartiles and medication status, particularly antihypertensive drugs and statin use, with significant interaction *P*-values. In the forest plot, the association appeared stronger in some age strata, while it was attenuated (or even reversed in direction) among medication users in certain strata (e.g., antihypertensive drug users and statin users), highlighting a clinically plausible pattern: medication status may reflect confounding by indication (those treated may differ substantially in baseline cardiometabolic risk), treatment effects on intermediate phenotypes, and/or differential risk-factor control that changes how CMI maps onto a chart-based risk classification. Interaction plots were consistent with these subgroup signals, further supporting that CMI's association with the risk category may depend on age and treatment context. These findings should be interpreted cautiously, but they also provide a meaningful direction for future work—particularly to clarify whether treatment modifies the mapping between CMI and estimated risk, or whether the observed heterogeneity is primarily due to confounding and compositional differences across strata.

### Machine-learning triangulation

To complement conventional regression, we implemented an interpretable ML framework (LASSO-random forest-SHAP) using a clinically accessible candidate set and intentionally excluding highly overlapping lipid metrics (e.g., triglycerides and non-HDL-C) to reduce redundancy because CMI already incorporates lipid-related and anthropometric information. In this framework, LASSO retained seven predictors, and a random forest model trained on these features achieved good discrimination (AUC: 0.814). Crucially for interpretability, SHAP ranking showed that CMI was ranked 3rd among the 7 LASSO-selected predictors (rank 3/7). This ranking supports that CMI contributes nontrivially to the model's predicted probability of being classified as WHO-defined high risk, even when considered alongside conventional factors.

That said, ML outputs should be interpreted as model-based attribution, not causal ordering. SHAP describes feature contribution within the trained model under the chosen data split and hyperparameters, and it is most useful here as a triangulation tool: it corroborates that CMI remains informative in a parsimonious predictor set rather than replacing epidemiologic inference.

### Clinical and public-health implications

Because CMI is derived from routinely available measurements (waist-to-height ratio and fasting triglycerides/HDL-C), it is feasible for large-scale community screening settings. Its association with WHO chart–defined high-risk status suggests that CMI may help identify individuals who warrant more comprehensive risk evaluation, counseling, or targeted preventive strategies, especially where resources constrain full risk workups. At the same time, given the modest discrimination of CMI alone, it is best positioned as an adjunct marker for screening and risk communication rather than a substitute for established risk charts.

### Future directions

Several next steps would strengthen clinical translation: (1) prospective validation against incident CVD events and comparison of incremental value beyond WHO charts; (2) external validation of the proposed cutoff and exploration of whether the optimal threshold differs by age, sex, and treatment strata; (3) deeper assessment of medication-related heterogeneity (including confounding by indication) using approaches such as propensity methods or sensitivity analyses; and (4) evaluation of whether CMI improves reclassification in pragmatic screening pathways.

### Strengths and limitations

This study has several strengths. First, it leveraged a large community-based screening dataset from the ChinaHEART program in Luohe, reflecting a real-world primary prevention setting with standardized field procedures and routine clinical measurements, which enhances the applicability of the findings to population screening. Second, we evaluated CMI comprehensively using complementary analytic approaches: (i) ROC analysis to derive a data-driven cutoff for pragmatic grouping; (ii) stepwise-adjusted logistic models to assess robustness under different confounding-control frameworks; (iii) restricted cubic splines to examine potential nonlinearity; and (iv) subgroup and formal interaction testing to explore effect heterogeneity across prespecified strata. Finally, we triangulated conventional epidemiologic results with an interpretable machine-learning workflow (LASSO for feature selection, random forest for classification, and SHAP for transparent attribution), which provided an additional perspective on the relative contribution of CMI within a parsimonious predictor set.

Several limitations merit consideration. First, the cross-sectional design precludes causal inference and does not allow temporal ordering between CMI and risk classification. Second, the outcome was WHO chart–defined CVD high-risk status rather than adjudicated CVD events; therefore, findings primarily reflect association with a risk-stratification category and may not directly translate to incident CVD without prospective validation. Third, although we used stepwise adjustment and prespecified covariates, residual confounding remains possible (e.g., diet, physical activity, socioeconomic factors beyond education, and other unmeasured cardiometabolic determinants). Fourth, medication use–stratified subgroup/interaction findings (notably antihypertensive drugs and statins) should be interpreted cautiously because medication status may introduce confounding by indication, reflect treatment effects, and in some strata the sample size may be relatively small, leading to unstable estimates. Fifth, the ROC-derived cutoff (Youden-based) was obtained from the same dataset and may be sample-dependent; external validation is needed before generalizing a single threshold to other populations or settings. Sixth, complete-case requirements and missing data may introduce selection bias; while we excluded participants with insufficient information for exposure/outcome/covariates, the excluded individuals may differ systematically from those retained. Finally, machine-learning results were intended to support interpretability and robustness rather than confirm causality; performance estimates may vary with different data splits or hyperparameters, and SHAP rankings describe contribution within the selected model rather than a universal causal ordering of predictors.

## Conclusion

CMI was positively associated with WHO-defined CVD high-risk status in a community screening population and showed a significant nonlinear dose–response relationship. Although discrimination of CMI alone was modest, CMI may serve as a simple, low-cost marker integrating dyslipidemia and central adiposity to support CVD risk stratification in primary prevention settings.

## Data Availability

The raw data supporting the conclusions of this article will be made available by the authors, without undue reservation.
